# *GmMYB21a* Improves Male Fertility of CMS-Based Restorer Line Under High-Temperature Stress in Soybean

**DOI:** 10.3390/plants15071040

**Published:** 2026-03-27

**Authors:** Jilei Gan, Hongjie Wang, Yujuan Gu, Xianlong Ding, Shouping Yang

**Affiliations:** National Innovation Platform for Soybean Breeding and Industry-Education Integration, Key Laboratory of Biology and Genetics and Breeding for Soybean, Ministry of Agriculture and Rural Affairs of the People’s Republic of China, Jiangsu Key Laboratory of Soybean Biotechnology and Intelligent Breeding, Zhongshan Biological Breeding Laboratory (ZSBBL), State Key Laboratory of Crop Genetics & Germplasm Enhancement and Utilization, Jiangsu Collaborative Innovation Center for Modern Crop Production, National Center for Soybean Improvement, College of Agriculture, Nanjing Agricultural University, Nanjing 210095, China

**Keywords:** soybean (*Glycine max* (L.) Merr.), *GmMYB21a*, high-temperature stress, male fertility, reactive oxygen species

## Abstract

High-temperature (HT) stress during flowering causes male sterility and yield loss in soybean. MYB transcription factors are key regulators under abiotic stress, yet their function and mechanism in regulating male fertility under HT stress in soybean are not fully understood. In this study, a MYB transcription factor *GmMYB21a* in soybean was identified. *GmMYB21a* was induced by HT stress in soybean restorer line and was specifically expressed in pollen. Through overexpression and knockout experiments, we demonstrated that *GmMYB21a* positively regulated pollen viability and germination under HT stress. Overexpression of *GmMYB21a* significantly enhanced these traits in restorer line, whereas knockout plants exhibited the opposite effect. Transcriptome sequencing revealed that *GmMYB21a* overexpression upregulated numerous stress-responsive genes, particularly those involved in flavonoid biosynthesis and sugar metabolism. In addition, molecular experiments confirmed that GmMYB21a bound to the promoter of flavonoid synthesis gene *GmCHI2-A* and promoted its expression. In summary, our research indicated *GmMYB21a* enhanced the HT-tolerance of male fertility in soybean restorer line through reactive oxygen species scavenging and flavonoid synthesis. This study aims to elucidate the thermotolerance mechanism in soybean male fertility and identify genetic resources for breeding HT-tolerant restorer lines.

## 1. Introduction

Soybean (*Glycine max* (L.) Merr.) is a globally cultivated crop and a primary source of vegetable oil and protein. Heterosis utilization has been demonstrated to be a promising strategy for enhancing soybean yield [1]. To date, heterosis utilization based on cytoplasmic male sterility (CMS) system have been established in major crops, including rice [2], maize [3], and rapeseed [4], making significant contributions to agricultural productivity [5]. China maintains a pioneering position in heterosis utilization research, particularly in cereal crop improvement. With the advancement of key technologies for the industrialization of hybrid soybean, the potential of heterosis is being further explored, showing broad application prospects along with economic and social benefits [6]. So far, more than 46 hybrid soybean varieties had been bred and approved in China, and the cultivation of hybrid soybean is still underway [1]. However, it had been reported that the male fertility of some CMS-based F_1_ combinations is highly sensitive to high-temperature (HT) stress. HT stress can induce male sterility, ultimately reducing seed production [7,8]. The primary reason for this was the difference in HT tolerance among the CMS-based restorer line parents under the same CMS line background [7,9,10], which may be attributed to specific molecular mechanisms affecting their stress response. Understanding the regulatory mechanisms of male fertility under HT stress in soybean CMS restorer lines is pivotal for uncovering genetic resources and, ultimately, developing breeding strategies to enhance HT-tolerance in hybrid soybeans. Prior work has established that in soybean, miR156b targeted GmSPL2b to regulates male fertility in CMS-based restorer lines under HT stress by modulating flavonoid metabolism [11]. However, our understanding of the regulatory mechanisms governing male fertility in these restorer lines under HT stress is still limited.

In plants, the MYB family represents one of the largest and most functionally diverse transcription factor (TF) families [12]. Within this family, the R2R3-type MYBs represent the largest subgroup [13]. Notably, R2R3-type MYB TFs are crucially implicated in both male reproductive development and the response to HT stress [14]. Studies in *Arabidopsis* demonstrated that AtMYB24 targets *AtCKL2* and *AtCKL7* to modulate their expression in early anthers under HT stress [15]. In rice, overexpression of *OsMYB55* conferred significant HT tolerance by upregulating the accumulation of both total and specific individual amino acids [16]. Additionally, the *pl* mutant in rice, regulated by the MYB TF *OsPL*, exhibited stronger HT resistance than the wild type during seed germination, which is associated with elevated levels of endogenous plant hormones [17]. Despite their known functions, the precise molecular functions of MYB TFs in governing male fertility under HT stress have not been fully elucidated.

As a major group of plant-derived secondary metabolites, flavonoids are prized and extensively researched for their ability to act as antioxidants and scavenge free radicals [18]. The production of major flavonoid classes—including chalcones, flavones, flavonols, anthocyanins, and proanthocyanidins—is catalyzed by a series of essential enzymes. Key enzymes involved in this pathway are chalcone synthase (CHS), chalcone isomerase (CHI), flavanone 3-hydroxylase (F3H), flavone synthase (FNS), flavonol synthase (FLS), and anthocyanidin synthase (ANS) [18,19]. Accumulating evidence underscores the crucial roles flavonoids play in plant stress responses [20,21]. For instance, enhanced abiotic stress tolerance can be mediated by UDP-glucosyltransferase *GSA1*. This enzyme confers tolerance by redirecting metabolic flux from lignin production to the biosynthesis of flavonoids [22]. Additionally, flavonols can protect spores and pollen against HT stress by scavenging reactive oxygen species (ROS) [23]. The biosynthesis of flavonoids in higher plants is subject to transcriptional control by a conserved transcriptional complex comprising MYB, bHLH, and WD40 proteins (the MBW complex) [24]. For example, in *Chrysanthemum*, *CmMYB012* was demonstrated to regulate *CmFNS* to inhibit flavone production and downregulate anthocyanin synthesis gene under HT stress [25]. In tea leaves, the CsMYB75/86-TT8-TTG1 complex regulates anthocyanin and catechin biosynthesis, whereas CsMYBL2, activated under HT stress, inhibits the formation of this complex and the production of anthocyanin/catechin [26].

Our previous study found that a MYB TF, *GmMYB305*, was up-regulated in the HT-tolerant NF_1_ compared with the HT-sensitive YF_1_ under HT stress in soybean [7]. We speculated that *GmMYB305* might confer HT tolerance in soybean. Based on latest gene website annotation “transcription factor MYB21-related”, homologous alignment results below and the existence of homologous genes in soybean, we renamed it as *GmMYB21a*. In this study, we found *GmMYB21a* was specifically expressed in pollen and respond to HT stress. Functional analysis of *GmMYB21a* revealed that *GmMYB21a* acts as a positive regulator of male fertility under HT stress through ROS scavenging in both the soybean restorer line and its hybrid F_1_. Additionally, based on transcriptome sequencing between *GmMYB21a-OE2* and TL1, we analyzed the expression and promoters of many stress-related differentially expressed genes. Further molecule experiments confirmed that *GmMYB21a* functioned within the flavonoid biosynthesis pathway, specifically by binding to the MBS motif of *GmCHI2-A* promoter. Our results provide preliminary evidence that *GmMYB21a* plays a role in mediating male fertility under HT stress, providing candidate gene resources for breeding HT-tolerant restorer lines in soybean.

## 2. Results

### 2.1. Characterization of Soybean GmMYB21a

**Figure 1:**
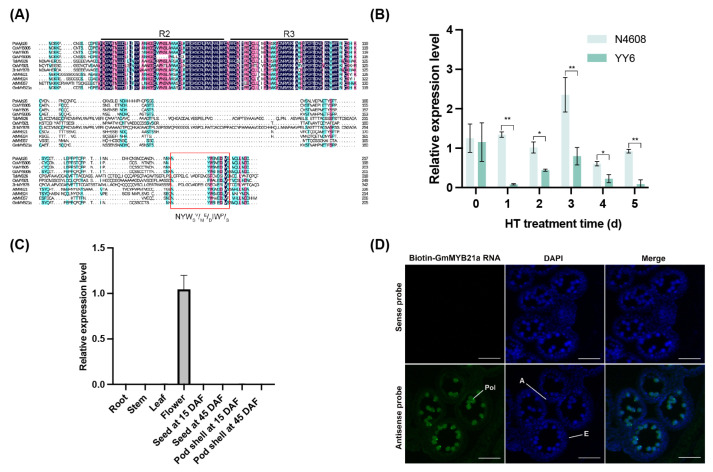
Characteristic analysis of *GmMYB21a*. (**A**) Multiple sequence alignment of GmMYB21a and its homologs other MYB family proteins from different species. The R2R3 conserved domains were marked by black line. Conserved NYWSV/ME/DIWP/S motifs were marked by red rectangle. (**B**) The relative expression levels of *GmMYB21a* in soybean high-temperature (HT)-tolerant restorer line N4608 and HT-sensitive restorer line YY6 under HT stress via reverse transcription quantitative real-time polymerase chain reaction (qRT-PCR) analysis. Asterisk indicates significant differences (*, *p* < 0.05; **, *p* < 0.01). (**C**) Tissue expression analysis of *GmMYB21a*. DAF, days after flowering. (**D**) RNA in situ hybridization of *GmMYB21a*. The sense probe was the control. DAPI, 4′,6-diamidino-2-phenylindole; A, Anther; Pol, Pollen; E, Epidermis layer. Bar, 50 μm.

Multiple sequence alignment revealed that GmMYB21a consisted of 206 amino acids, and similar to homologous proteins in other species, contained the conserved R2R3 domain and the NYW_S_^V^/_M_^E^/_D_IW^P^/_S_ motif (Figure 1A). Neighbor-joining phylogenetic analysis showed that GmMYB21a shares closest homology with GsMYB305 from *Glycine soja*, AtMYB21, and AtMYB24 both from *Arabidopsis* (Supplementary Figure S1). The transactivation assay in yeast revealed that GmMYB21a possessed transactivation activity, as evidenced by its ability to activate the *MEL1*, *ADE2*, and *HIS3* reporter genes. Furthermore, deletion or mutation of the C-terminal domain completely abolished GmMYB21a’s transactivation activity (Supplementary Figure S2). qRT-PCR analysis revealed a significant upregulation of *GmMYB21a* in HT-tolerant restorer line “N4608” than that in HT-sensitive restorer line “YY6” under HT stress at 1–5 d (Figure 1B). Tissue expression analysis by qRT-PCR showed the specific expression of *GmMYB21a* in soybean flowers (Figure 1C). Further RNA in situ hybridization analysis revealed that *GmMYB21a* mRNA was predominantly localized in soybean pollen (Figure 1D). Subcellular localization assay showed that the fluorescent signal of GmMYB21a was detected in the nucleus, overlapping with nuclear marker (Supplementary Figure S3).

### 2.2. GmMYB21a Positively Regulates Male Fertility of Restorer Line Under HT Stress

**Figure 2:**
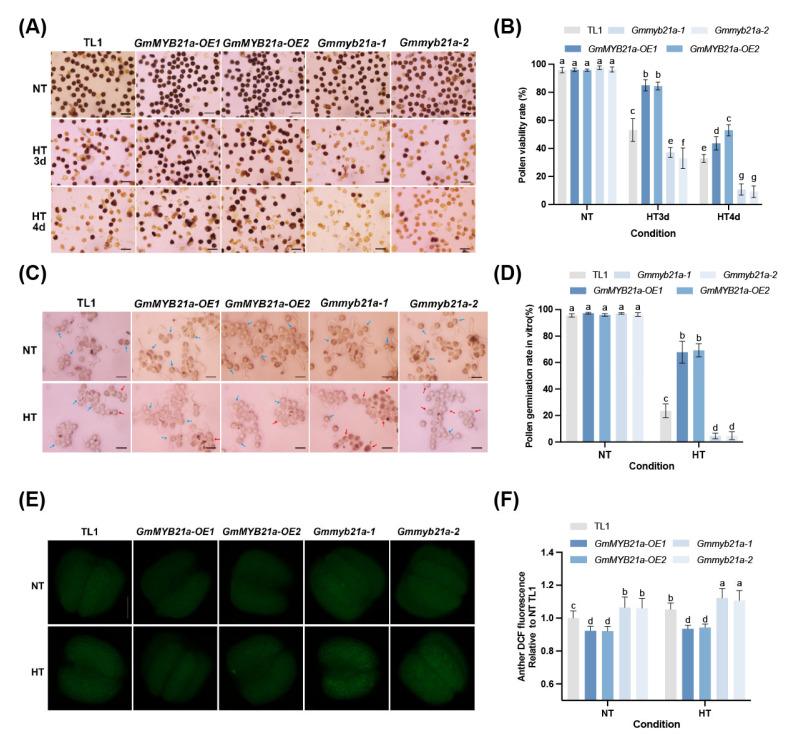
*GmMYB21a* positively regulates male fertility of restorer line under HT stress (**A**) Pollen I_2_-KI staining of *GmMYB21a-OE1*, *GmMYB21a-OE2*, *Gmmyb21a-1*, *Gmmyb21a-2*, and Tianlong No.1 (TL1) under HT stress. (**B**) Pollen viability rate statistics (**C**) Pollen germination on the pollen germination medium of *GmMYB21a-OE1*, *GmMYB21a-OE2*, *Gmmyb21a-1*, *Gmmyb21a-2*, and TL1 under HT stress. The blue arrows indicate normally germinated pollen, while the red arrows indicate pollen that cannot germinate normally. (**D**) Pollen germination rate statistics. (**E**) 2′-7′-dichlorodihydrofluorescein diacetate (CM-H_2_DCFDA) staining of anther of *GmMYB21a-OE1*, *GmMYB21a-OE2*, *Gmmyb21a-1*, *Gmmyb21a-2*, and TL1 under HT stress. (**F**) DCF fluorescence statistics of anthers. Different lowercase letters indicate significant differences (Duncan’s test, *p* < 0.05). Bar, 50 μm in (**A**,**C**), 200 μm in (**E**).

To clarify the functional role of *GmMYB21a*, two *GmMYB21a* overexpression lines *GmMYB21a-OE1* and *GmMYB21a-OE2* were obtained with the recipient Tianlong No. 1 (TL1, a CMS-based restorer line) using pTF101.1 vector (Supplementary Figure S4A). Meanwhile, two homozygous single mutants *Gmmyb21a-1* and *Gmmyb21a-2* were generated by CRISPR/Cas9 system, which carrying a 10-bp deletion and a 1-bp insertion, respectively. Both mutations resulted in a frameshift and premature termination of GmMYB21a translation (Supplementary Figure S4B,C). We selected *GmMYB21a-OE1*, *GmMYB21a-OE2*, *Gmmyb21a-1*, *Gmmyb21a-2* and TL1 for 5 d HT stress. Under NT condition, the pollen viability rates of *GmMYB21a-OE1*, *GmMYB21a-OE2*, *Gmmyb21a-1*, *Gmmyb21a-2*, and TL1 were 96.1%, 95.8%, 97.5%, 96.3%, and 95.9% respectively, with no significant differences (Figure 2A,B). After 3 d of HT stress, the pollen viability rate of TL1 rapidly declined to 53.1%. In contrast, *GmMYB21a-OE1* and *GmMYB21a-OE2* were less affected by HT stress, maintaining pollen viability rates above 80%. *Gmmyb21a-1* and *Gmmyb21a-2* were greatly affected by HT stress, with pollen viability rates sharply dropping below 40%. Following 4 d of HT stress, *GmMYB21a-OE1* and *GmMYB21a-OE2* still retained approximately half of their viable pollen, with pollen viability rates of 43.6% and 52.9%, respectively, significantly higher than that of TL1 (32.8%). In contrast, only about 10% of the pollen from *Gmmyb21a-1* and *Gmmyb21a-2* could be stained by I_2_-KI, which is significantly less than TL1 (Figure 2A,B).

Subsequently, an in vitro pollen germination assay under HT stress was performed. Under NT condition, the pollen from *GmMYB21a-OE1*, *GmMYB21a-OE2*, *Gmmyb21a-1*, *Gmmyb21a-2*, and TL1 germinated normally, with pollen germination rates of 97.2%, 95.9%, 97.1%, 96.1%, and 95.6% respectively (Figure 2C,D). After HT stress, all materials exhibited pollen that either failed to germinate or showed abnormal pollen tube elongation. In particular, the pollen of *Gmmyb21a-1* and *Gmmyb21a-2* almost failed to germinate, with pollen germination rates of only 4.5% and 4.7%, respectively. However, *GmMYB21a-OE1* and *GmMYB21a-OE2* maintained approximately 68% normal pollen germination and elongation, which was significantly higher than the 23.5% observed in TL1 (Figure 2C,D).

ROS are important signaling molecules. The dynamic balance between their production and scavenging orchestrates abiotic stress responses [27,28,29,30]. Therefore, we qualitatively evaluated ROS levels in the anthers of all soybean materials using 2′,7′-dichlorodihydrofluorescein diacetate (CM-H_2_DCFDA) staining. Clearly, compared to TL1, we detected significantly more ROS fluorescence in *Gmmyb21a-1* and *Gmmyb21a-2*, indicating that more ROS accumulated in *Gmmyb21a-1* and *Gmmyb21a-2* under NT condition (Figure 2E,F). Conversely, only weak ROS fluorescence was observed in *GmMYB21a-OE1* and *GmMYB21a-OE2*, with ROS levels significantly lower than in TL1. After HT stress, ROS levels were significantly elevated in *Gmmyb21a-1*, *Gmmyb21a-2*, and TL1. Notably, the two mutant lines *Gmmyb21a-1* and *Gmmyb21a-2* accumulated markedly higher levels of ROS compared to TL1. However, *GmMYB21a-OE1* and *GmMYB21a-OE2* still showed weak ROS fluorescence and maintained a significantly low level of ROS than TL1 (Figure 2E,F).

### 2.3. GmMYB21a Positively Regulates Male Fertility of Hybrid F_1_ Under HT Stress

**Figure 3:**
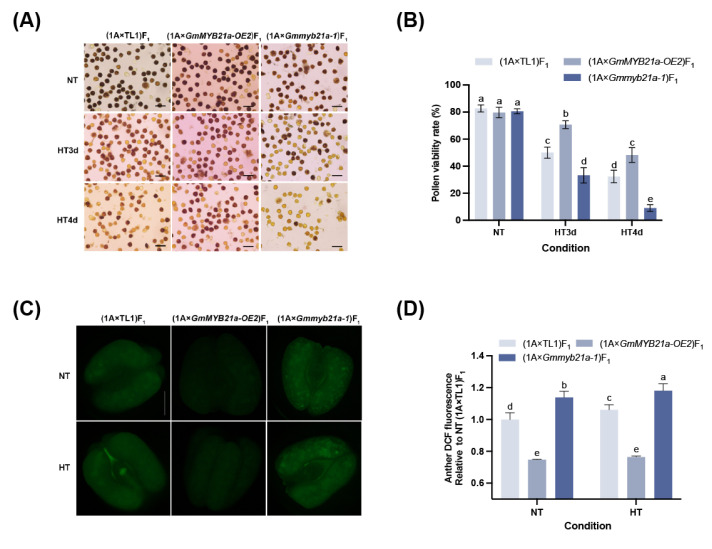
*GmMYB21a* positively regulates male fertility of hybrid F_1_ under HT stress. (**A**) Pollen I_2_-KI staining of (1A × TL1)F_1_, (1A × *GmMYB21a-OE2*)F_1_, and (1A × *Gmmyb21a-1*)F_1_ under HT stress. (**B**) Pollen viability rate statistics. (**C**) CM-H_2_DCFDA staining of anther of (1A × *GmMYB21a-OE2*)F_1_, (1A × *Gmmyb21a-1*)F_1_, and (1A × TL1)F_1_ under HT stress. (**D**) DCF fluorescence statistics. Different lowercase letters indicate significant differences (Duncan’s test, *p* < 0.05). Bar, 50 μm in (**A**), 200 μm in (**C**).

In order to investigate whether *GmMYB21a* is involved in the regulation of male fertility of hybrid F_1_ under HT stress, CMS line NJCMS1A was crossed with TL1, GmMYB21a-OE2 and Gmmyb21a-1 respectively and thus (1A × TL1)F_1_, (1A × *GmMYB21a-OE2*)F_1_ and (1A × *Gmmyb21a-1*)F_1_ were obtained. We further analyzed the male fertility of three hybrid F_1_ under HT stress. Under NT condition, the pollen viability rates of (1A × *GmMYB21a-OE2*)F_1_, (1A × *Gmmyb21a-1*)F_1_, and (1A × TL1)F_1_ were 79.6%, 80.5%, and 82.6%, respectively, with no significant differences observed (Figure 3A,B). After 3 d of HT stress, (1A × *GmMYB21a-OE2*)F_1_ maintained 70.7% viable pollen, which significantly more than TL1 (32.4%). In contrast, (1A × *Gmmyb21a-1*)F_1_ only kept 33.2% viable pollen. Following 4 d of HT stress, approximately half of the pollen in (1A × *GmMYB21a-OE2*)F_1_ was still stained, with a pollen viability rate of 48.2%, significantly higher than (1A × TL1)F_1_. Only 9% viable pollen could be observed in (1A × *Gmmyb21a-1*)F_1_, which significantly fewer than TL1 (Figure 3A,B). The differences in pollen viability rate among F_1_ under HT stress were attributed to variations in HT tolerance of the respective CMS-based restorer line. Further CM-H_2_DCFDA staining results showed that (1A × *GmMYB21a-OE2*)F_1_ exhibited a significantly lower ROS level, while (1A × *Gmmyb21a-1*)F_1_ displayed a significantly higher ROS level compared to (1A × TL1)F_1_ under HT stress (Figure 3C,D).

### 2.4. GmMYB21a Affects Flavonoid Biosynthesis and Stress Response Under HT Stress

**Figure 4:**
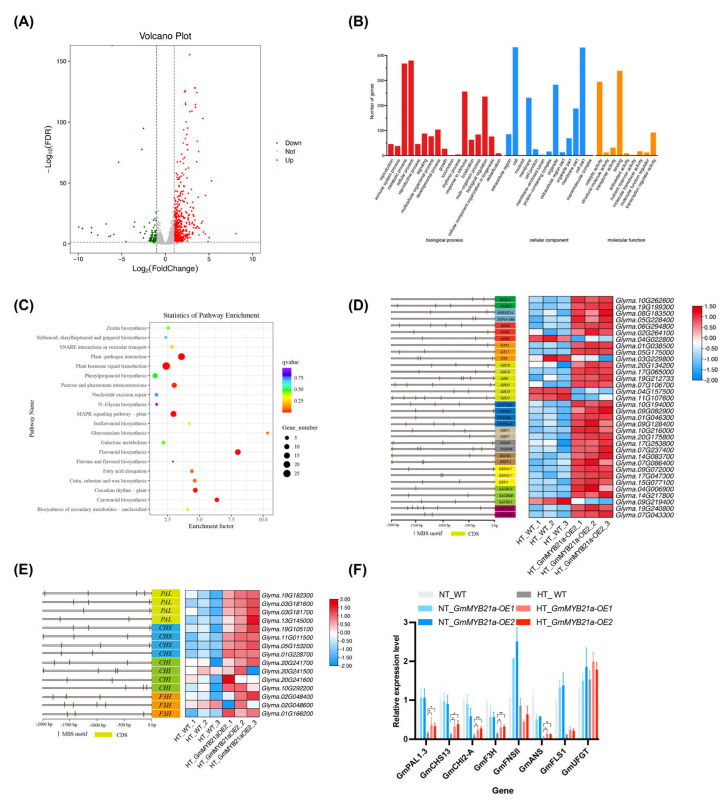
Comparative transcriptome analysis between *GmMYB21a-OE2* and TL1 under HT stress. (**A**) Volcano Plot of differentially expressed genes (DEGs) between *GmMYB21a-OE2* and TL1. The vertical dashed lines were set at ∣Log_2_(FoldChange)∣ = 1, and the horizontal dashed line was set at −Log_10_(FDR) = 0 (corresponding to the significance threshold of FDR = 0.05) (**B**) Gene ontology (GO) analysis of DEGs between *GmMYB21a-OE2* and TL1. (**C**) Kyoto encyclopedia of genes and genomes (KEGG) analysis of DEGs between *GmMYB21a-OE2* and TL1. (**D**,**E**) Promoter architecture and expression levels of stress-responsive DEGs. (**F**) Validation of DEGs related to flavonoid biosynthesis by qRT-PCR. Asterisks denote significant differences (*, *p* < 0.05; **, *p* < 0.01).

A comparative transcriptome analysis of flower buds from *GmMYB21a-OE2* and its recipient TL1 under HT stress was conducted. Compared to TL1, the *GmMYB21a-OE2* exhibited 666 significantly up-regulated and 147 significantly down-regulated genes, totaling 813 differentially expressed genes (DEGs) (Figure 4A). Gene ontology (GO) analysis indicated that the DEGs were primarily enriched in the categories of catalytic activity, cell part, and metabolic process (Figure 4B). Kyoto encyclopedia of genes and genomes (KEGG) analysis revealed that the DEGs were primarily associated with flavonoid biosynthesis and signaling pathways transduction (Figure 4C). Further analysis showed that several DEGs in *GmMYB21a-OE2* were associated with the ubiquitination process, including the E3 ubiquitin ligase *PUB22* (Figure 4D). In addition, genes involved in sugar metabolism and transport were up-regulated, including *sugar transporter 14* (*STP14-like*), *sucrose transporter 24* (*SWEET24*), *GDSL esterases* (*GDSLs*), *glycosyltransferases* (*GTs*), *glycoside hydrolases* (*GHs*), and *UDP-glucosyltransferases* (*UGTs*). Moreover, genes associated with flavonoid biosynthesis, phytohormone signaling, and stress response exhibited up-regulation in *GmMYB21a-OE2* compared to TL1, such as *phenylalanine ammonia-lyases* (*PALs*), *gibberellin-regulated proteins* (*GRPs*) and *heat shock transcription factors* (*HSFs*) (Figure 4E). Above analysis suggested that the up-regulation of genes related to flavonoid biosynthesis, phytohormone signaling, and stress response contributed to the improved male fertility of *GmMYB21a* overexpression lines under HT stress. We selected eight flavonoid biosynthesis-related genes for qRT-PCR validation and the results agreed with the RNA-Seq data (Figure 4F).

### 2.5. GmMYB21a Directly Binds to the GmCHI2-A Promoter

**Figure 5:**
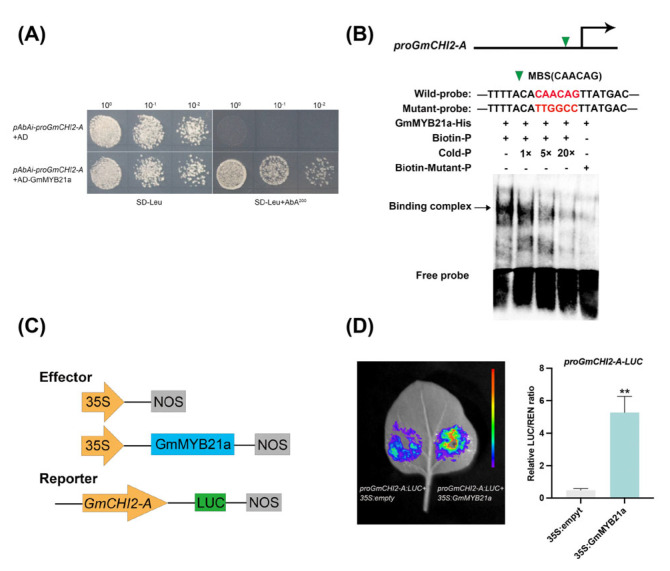
GmMYB21a activates the transcription of *GmCHI2-A*. (**A**) GmMYB21a bound to the promoter of *GmCHI2-A* through yeast one-hybrid (Y1H) assay. AD-Empty+*pABAi-proGmCHI2-A* were used as the negative control. Aureobasidin A, AbA. (**B**) The binding of GmMYB21a to the MBS motif (CAACAG) of *GmCHI2-A* promoter verified by electrophoretic mobility shift assay (EMSA). The wild-type probe sequence containing the MBS motif was “TTTTACACAACAGTTATGAC”. When the MBS motif was mutated, the sequence became “TTTTACATTGGCCTTATGAC”. (**C**) Schematic of the effectors and reporters for the dual-luciferase assay. The yellow arrows represented different promoters, the blue and green rectangles represented coding sequences, and the gray rectangles represented the terminator. (**D**) Transcriptional activity of *proGmCHI2-A* activated by *GmMYB21a* in *Nicotiana benthamiana* (*N. benthamiana*) leaves (**, *p* < 0.01).

Promoter element analysis showed that the promoters of these genes contained at least one MYB binding site (MBS) (Figure 4E). Compared with TL1, the expression levels of flavonoid biosynthesis-related DEGs, including *GmPAL1.3*, *GmCHS13*, *GmCHI2-A*, *GmF3H*, and *GmANS*, were significantly elevated in *GmMYB21a-OE2*, suggesting that GmMYB21a activate their expression (Figure 4F). Yeast one-hybrid (Y1H) assay revealed that yeast cells co-transformed with *GmMYB21a* and *proGmCHI2-A* were able to grow on medium containing Aureobasidin A, confirming the binding of GmMYB21a to the *GmCHI2-A* promoter (Figure 5A). Further electrophoretic mobility shift assay (EMSA) revealed that HIS-tagged GmMYB21a specifically bound to the CAACAG motif in vitro, with binding ability gradually decreasing with the increment of cold probe. Mutation of the conserved motifs abolished the formation of protein-DNA complexes (Figure 5B). Moreover, dual-luciferase assay showed that GmMYB21a significantly activated the expression of *GmCHI2-A*, by observing the stronger luciferase fluorescence (Figure 5C,D). Collectively, these findings indicated that GmMYB21a target to *GmCHI2-A* to activate its expression.

### 2.6. GmMYB21a Participates in Kaempferol Biosynthesis

**Figure 6:**
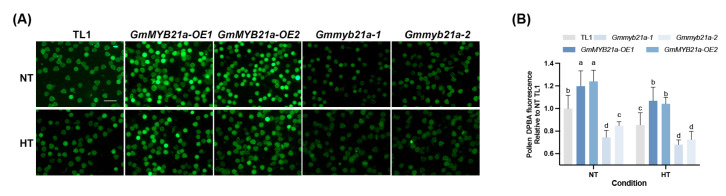
*GmMYB21a* affects kaempferol synthesis in pollen of soybean restorer line under HT stress. (**A**) Diphenylboric acid 2-amino ethyl ester (DPBA) staining of pollen of *GmMYB21a-OE1*, *GmMYB21a-OE2*, *Gmmyb21a-1*, *Gmmyb21a-2*, and TL1 under HT stress. Bar, 100 μm. (**B**) DPBA fluorescence statistics of pollen. Different lowercase letters indicate significant differences (Duncan’s test, *p* < 0.05).

Flavonoids, as potent ROS scavengers, may therefore play a crucial protective role against HT stress [31,32,33]. We subsequently qualitatively detected the kaempferol levels in soybean pollen through diphenylboric acid 2-aminoethyl ester (DPBA) staining. Interestingly, due to the overexpression of *GmMYB21a*, most pollen grains in *GmMYB21a-OE1* and *GmMYB21a-OE2* exhibited higher fluorescence intensity compared to TL1 under normal condition, indicating a greater content of kaempferol. Conversely, the fluorescence intensity and kaempferol content of pollen grains in *Gmmyb21a-1* and *Gmmyb21a-2* were significantly lower than in TL1 due to the knockout of *GmMYB21a* (Figure 6A,B). Under HT stress, kaempferol synthesis in all materials was inhibited. Similarly, *GmMYB21a-OE1* and *GmMYB21a-OE2* exhibited significantly higher kaempferol levels compared to TL1, while significantly less kaempferol content was detected in *Gmmyb21a-1* and *Gmmyb21a-2* (Figure 6A,B).

## 3. Discussion

HT stress is a major environmental constraint that compromises crop production. Researchers have extensively investigated how plants respond to HT stress in recent decades [34,35]. Soybean, as a key rich source of vegetable protein and oil, exhibits significant yield advantages in hybrid varieties. However, certain CMS-based F_1_ combinations in soybean show high sensitivity to environmental factors, particularly HT stress [11]. Previous studies have found that improving the restorability or HT-tolerance in restorer lines can effectively enhance HT tolerance of male fertility in soybean and cotton hybrid F_1_, in which HT tolerance and fertility-enhancing genes playing key roles [11,36,37]. Therefore, identifying genes that improve both HT tolerance and male fertility, and elucidating their regulatory mechanisms under HT stress, is of great importance. By introducing HT tolerance and fertility-enhancing genes into restorer lines, it is conceivable to engineer HT-tolerant, strong restorers. Such lines would enhance the male fertility of hybrid F_1_ plants under high temperatures, thereby paving the way for breeding novel hybrid soybean varieties with stable fertility.

In plants, the MYB transcription factor family represents a major group of transcriptional regulators characterized by remarkable functional diversity [12]. Numerous MYB transcription factors have been found in different crops, including soybean [38], *Arabidopsis* [39], rice [40], and tomato [41]. Our previous study identified *GmMYB21a* as a candidate gene conferring HT tolerance in the soybean CMS-based restorer line [7]. It has been established in *Arabidopsis* and lily that *AtMYBS1* and *LlMYB305* expression is induced in response to HT stress [42,43]. Similarly, the expression of *GmMYB21* was also found to be up-regulated by HT stress. The specificity of gene expression location implies particular functions. For example, the MYB protein Baymax1 is preferentially expressed during stages 5 to 10 of rice anther development and is one of the key regulators of male fertility in rice [44]. In addition, pepper R2R3-MYB transcription factor *Capana10g000198* is an anther-specific gene. Downregulation of *Capana10g000198* result in male sterility [45]. Our study showed specific expression of *GmMYB21a* in soybean pollen (Figure 1D), implying its function in regulating male fertility. Previous studies have indicated that MYB transcription factors act as either transcriptional activators or repressors. For instance, under HT stress, StMYB44 acts as a repressor of anthocyanin biosynthesis by directly binding to and suppressing the DFR promoter [46]. In contrast, a transactivation assay in yeast demonstrated that GmMYB21a exhibits strong transactivation activity, confirming its function as a transcriptional activator (Supplementary Figure S2).

A growing body of evidence has revealed that MYB transcription factors serve diverse biological functions, including roles in male reproductive development [47], biotic and abiotic stress [48], and secondary metabolite production [49]. Homologs of AtMYB21 have been confirmed to regulate anther dehiscence [50], low temperature flowering process [51], and linalool biosynthesis in flowers [52]. However, functional studies on AtMYB21 homologs regulating male fertility under HT stress remain rarely reported. Through functional analysis of *GmMYB21a* in soybean recipient TL1 using transgenic overexpression and the CRISPR-Cas9 system, we demonstrated that *GmMYB21a* positively regulates male fertility in the soybean CMS-based restorer line under HT stress (Figure 2A).

Flavonoids are defined as a class of polyphenolic metabolites featuring a C6-C3-C6 backbone and are best known for their significant antioxidative potential and effective radical scavenging [18]. Flavonols, a type of flavonoids, can reduce ROS in tomato pollen grain and pollen tube under elevated temperature, thereby alleviating the inhibition of pollen tube growth [31]. Further studies revealed that mutation of *FLAVANONE 3-HYDROXYLASE* (*F3H*) impairs flavonol antioxidant biosynthesis [32]. Kaempferol belongs to the flavonol subgroup of the flavonoid family. In this study, kaempferol levels in pollen of all soybean materials were qualitatively evaluated using DPBA staining. The results indicated that elevated kaempferol levels were positively associated with male fertility and inversely correlated with ROS levels (Figure 2 and Figure 6).

The MBW complex serves as a conserved regulator in the transcriptional control of flavonoid biosynthesis [24,53]. For instance, OsMYB30 up-regulated the expression of *OsPAL6* and *OsPAL8*, thereby altering lignin and salicylic acid biosynthesis in rice leaf sheaths [54]. Similarly, DzMYB2 regulated the expression of *PAL*, *CHS*, and *CHI*, affecting phenylpropanoid biosynthesis in durian pulps [55]. This study showed that the transcription factor *GmMYB21a* activates *GmCHI2-A* expression, thereby increasing kaempferol levels (Figure 5 and Figure 6). Previous studies on MYB-regulated flavonoid biosynthesis have primarily emphasized roles in plant disease resistance, salt or drought stress. Our findings, however, unveiled a new pathway through which a MYB transcription factor modulates flavonoid pathway to enhance HT tolerance of male fertility of restorer line in soybean.

## 4. Materials and Methods

### 4.1. Plant Materials and Growth Conditions

The soybean cultivar Tianlong No. 1 (TL1), a restorer line for the CMS line NJCMS1A, served as the control. The NJCMS1A [56,57] and its restorer lines N4608, YY6 [58], and TL1 were obtained from the National Center for Soybean Improvement in Nanjing, China. NJCMS1A exhibits sporophyte sterility [59], with cytological observations indicating that pollen abortion occurs at the binucleate stage [60].

All soybean plants were cultivated in a climate-controlled growth chamber (RXZ-430D, Ningbojiangnan, Ningbo, China). During the seedling stage, they were exposed to long-day photoperiods (16 h light:8 h dark), a temperature of 28 °C/22 °C (day/night), and 70% relative humidity. This was followed by a flowering stage under short-day photoperiods (12 h light:12 h dark) with the same conditions. For the HT treatment, soybean plants at the R1 stage were placed in a growth chamber set to 40 °C/34 °C (day/night) with a 12 h light/12 h dark photoperiod and 70% relative humidity for 5 d.

The *Nicotiana benthamiana* (*N. benthamiana*) were used for transient transformation. All *N. benthamiana* plants were cultivated in chambers under a 16 h/8 h (light/dark) photoperiod at 22 °C. For the HT treatment, plants were incubated at 40 °C for 3 h.

### 4.2. Subcellular Localization

The 35S::GFP (green fluorescent protein, GFP) and 35S::GmMYB21a-GFP fusion plasmids were independently transformed into *Agrobacterium tumefaciens* strain GV3101. The bacterial cultures were grown and centrifuged to collect the cells, which were resuspended in infiltration buffer. The leaves of 4-week-old *N. benthamiana* were separately injected with the resulting suspensions. After injection, the *N. benthamiana* plants were kept in darkness for 48 h. Subcellular localization was then examined by confocal microscopy (Zeiss LSM 780, Jena, Germany) of the injected leaf areas, and images were captured for analysis.

### 4.3. RNA Extraction and qRT-PCR Assay

Total RNA was extracted from flower buds of various sizes collected from the upper, middle, and lower parts of soybean plants, including *GmMYB21a* (*XP_003553814.1*) overexpression lines, TL1, N4608, and YY6, using RNAex Pro Reagent (Accurate biotechnology, Changsha, China). cDNA transcription was conducted by Evo M-MLV RT Kit (Accurate Biotechnology, Changsha, China), All experiments were performed with three biological replicates. The relative expression levels of genes were calculated via the 2^−ΔΔCT^ method [61], employing *GmActin11* as an internal control for normalization [62].

### 4.4. Yeast One-Hybrid (Y1H) Assay

Y1H assays were carried out with the Matchmaker™ Gold system (Clontech, CA, USA), following the manufacturer’s protocol. For the generation of bait construct, the GmCHI2-A promoter (*NP_001236768.2*) was amplified from soybean line N4608 genomic DNA and cloned into the pAbAi. For the generation of the prey construct, the *GmMYB21a* CDS was cloned into the pGADT7. The protein-DNA interactions were assessed on SD/-Leu medium supplemented with 200 mM AbA.

### 4.5. Dual-Luciferase Reporter Assay

The *GmCHI2-A* promoter and the *GmMYB21a* CDS were cloned into the pGreenII 0800-LUC and pGreenII 62-SK vectors to generate the reporter construct *proGmCHI2-A::LUC* and the effector construct *35S::GmMYB21a*, respectively. All recombinant plasmids were independently transformed into Agrobacterium tumefaciens strain GV3101 harboring the pSoup helper plasmid. The bacterial cultures were grown to an optical density (OD600) of approximately 0.6, harvested by centrifugation, and resuspended in infiltration buffer. For transient transformation, equal volumes of the reporter and effector bacterial suspensions were mixed and co-infiltrated into the leaves of 4-week-old *N. benthamiana*. After injection, the *N. benthamiana* plants were kept in darkness for 24 h followed by 24 h under normal light conditions. LUC fluorescence was detected using the NightSHADE LB 985 imaging system (Berthold, Black Forest, Germany). Firefly and renilla luciferase activities were detected using the Dual Luciferase Reporter Gene Assay Kit (Yeasen biotechnology, Shanghai, China) according to the manufacturer’s instructions.

### 4.6. Electrophoretic Mobility Shift Assay (EMSA)

The CDS of *GmMYB21a* was inserted into the pET32a vector, and then the recombinant construct was transformed into the *E. coli* BL21 (DE3). The fusion protein was expressed by induction with IPTG at 16 °C and purified using GST beads (Solarbio, Beijing, China). Biotin-labeled probes for EMSA were synthesized by General Biology Co., Ltd. (Anhui, China). The same unlabeled oligonucleotide severed as the competitor. The MBS motif (CAACAG) was replaced with TTGGCC as mutant probe. The EMSA was conducted using the EMSA Gel-Shift Kit (Beyotime, Shanghai, China).

### 4.7. RNA In Situ Hybridization

The flower buds of the restorer line N4608, which were expected to open the following morning, were collected in the afternoon and fixed in 50% FAA solution. Sample fixation and sectioning were performed according to a previous method [63]. A specific fragment of the *GmMYB21a* CDS was inserted into the pGEM-T Easy vector. The recombinant plasmid was linearized and served as the transcription template in vitro transcription. In situ probes were synthesized using a Biotin RNA Labeling Kit (Beyotime, Shanghai, China). Hybridization steps was conducted using a Biotin Fluorescence in Situ Hybridization Kit for RNA (Beyotime, Shanghai, China).

### 4.8. Male Fertility Investigation

For soybean pollen fertility observation in soybean, the flower buds from each material that were expected to open the following morning were collected in the afternoon. Anthers were gently crushed using tweezers to release pollen onto a glass slide, and the pollen grains were stained with 1% I_2_-KI solution. Staining results were observed, photographed, and counted using a microscope (Olympus CX31, Tokyo, Japan). For soybean pollen germination assay in vitro, opening flowers of each material were collected in the morning. Pollen grains were transferred to germination medium. For the control, they were treated with 30 °C for 0.5 h. Incubation was conducted with 40 °C for 0.5 h as HT stress treatment [64]. A pollen grain was scored as germinated when its pollen tube length exceeded its diameter [65]. Three biological replicates were set for each material, with two plants per biological replicate.

### 4.9. Staining of Anther and Pollen

To detecting ROS fluorescence in anther, *GmMYB21a* overexpression lines, *Gmmyb21a* mutants, and TL1 were subjected to HT stress for 1 d. Flower buds from each material that were expected to open the following morning were collected in the afternoon, and their calyx, flag, and wing petals were removed. Then all samples were stained in TBS containing 5 μM CM-H_2_DCFDA and 0.005% (*v*/*v*) Triton X-100 for 20 min at 28 °C [31]. Following staining, the TBS solution containing the CM-H_2_DCFDA probe was removed and replaced with fresh TBS. The stained anthers were subsequently mounted on a microscope slide for imaging using a fluorescence stereomicroscope. (Leica M165FC, Wetzlar, Germany).

For fluorescence detection of flavonoids in pollen, flower buds of each material were collected and stained in PBS containing 0.25% (*w*/*v*) DPBA and 0.005% (*v*/*v*) Triton X-100 for 2 h at 28 °C [33]. After staining, the PBS containing DPBA was replaced with fresh PBS, and anthers were dissected to release pollen onto a microscope slide for imaging by a fluorescent stereo microscope (Leica M165FC, Wetzlar, Germany). After laser excitation, the emission spectrum of the kaempferol-DPBA complex was collected within the wavelength range of 495–540 nm [66]. Quantification of both ROS and DPBA fluorescence intensity was performed using Fiji [67]. Three biological replicates were set for each material, with two plants per biological replicate.

### 4.10. RNA-Seq Analysis

The TL1 and *GmMYB21a-OE2* plants were subjected to HT stress during flowering. Flower buds from the upper, middle, and lower parts of TL1 and *GmMYB21a-OE2* plants were collected, respectively. Following sequencing by Genepioneer Biotechnologies Co., Ltd. (Nanjing, China), the raw reads were quality-trimmed and filtered to generate clean data. Differentially expressed genes (DEGs) were used for Gene Ontology (GO) and Kyoto Encyclopedia of Genes and Genomes (KEGG) pathway enrichment analyses. All analyses were performed with three independent biological replicates.

### 4.11. Statistical Analysis

Two groups differences were analyzed by Student’s *t*-test. For comparisons involving more than two groups, statistical significance was assessed using one-way ANOVA, with Duncan’s test (*p* < 0.05).

## 5. Conclusions

In summary, this study confirmed that *GmMYB21a*, a member of the MYB transcription factor family, acted as a positive regulator of male fertility under HT stress in soybean restorer line. Through functional analysis utilizing overexpression lines and knockout lines, we found that *GmMYB21a* overexpression enhanced male fertility under HT stress, while its deficiency increased the sensitivity of male fertility. The superior HT tolerance of *GmMYB21a*-overexpression plants was mechanistically linked to the antioxidant defense system, characterized by a significant reduction in ROS and increased accumulation of kaempferol in anthers and pollen, which maintains better anther growth and development under HT stress. Overall, our findings revealed the crucial role of *GmMYB21a* in regulating male fertility under HT stress, primarily through the modulation of ROS homeostasis and flavonoid biosynthesis. This work not only advanced the molecular understanding of HT stress signaling in soybean but also identified *GmMYB21a* as a promising target for genetic engineering and molecular breeding strategies aimed at developing restorer lines with stable male fertility.

## Data Availability

The RNA-seq raw data have been deposited in the NCBI SRA database with BioProject number PRJNA1356958. The all gene counts data has been deposited in the GEO database, with the accession number GSE325098.

## Supplementary Materials

The following supporting information can be downloaded at: https://www.mdpi.com/article/10.3390/plants15071040/s1, Figure S1: Phylogenetic analysis of GmMYB21a and related MYB proteins; Figure S2: Tissue expression analysis of *GmMYB21a*; Figure S3: Amino acid sequence alignment and phylogenetic analysis of GmMYB21a; Figure S4: Identification of *GmMYB21a* overexpression lines and *Gmmyb21a* mutants; Table S1: Primers used in this study; Table S2: Data for bar charts; Table S3: Data for volcano plots; Table S4: The expression counts and GO/KEGG annotations.
