# Supplementary material for: GmMYB21a Improves Male Fertility of CMS-Based Restorer Line Under High-Temperature Stress in Soybean
Source: Plants (Basel). 2026 Mar 27;15(7):1040. doi: 10.3390/plants15071040 (PMC13074783; doi:10.3390/plants15071040)
Supplement: Supplementary file 1 [file plants-15-01040-s001.zip › Supplementary Figures.pdf]

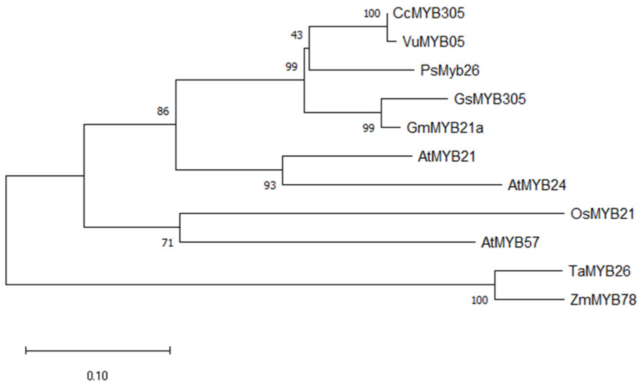

Supplementary Figure S1. Phylogenetic analysis of GmMYB21a and related MYB proteins.

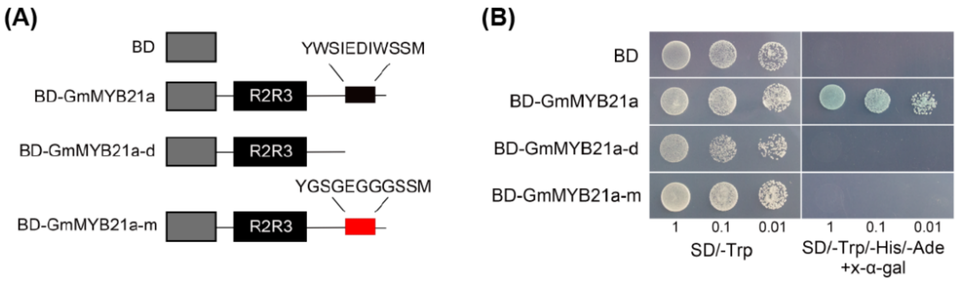

Supplementary Figure S2. Transactivation assay of GmMYB21a. (A) The vector construction used for the transactivation assay. The C-terminal of GmMYB21a was truncated or mutated for the transactivation assay. The black and red boxes indicated the activation motif and its mutant, respectively. BD, pGBKT7. (B) Transactivation activity of the different vectors. The SD/-Trp was used to detect transformants' growth and the SD/-Trp/-His/-Ade with x-α-gal was used to detect the transactivation activity. The 1, 0.1, and 0.01 represented the dilution concentration of yeast cells. SD, synthetic dropout medium.

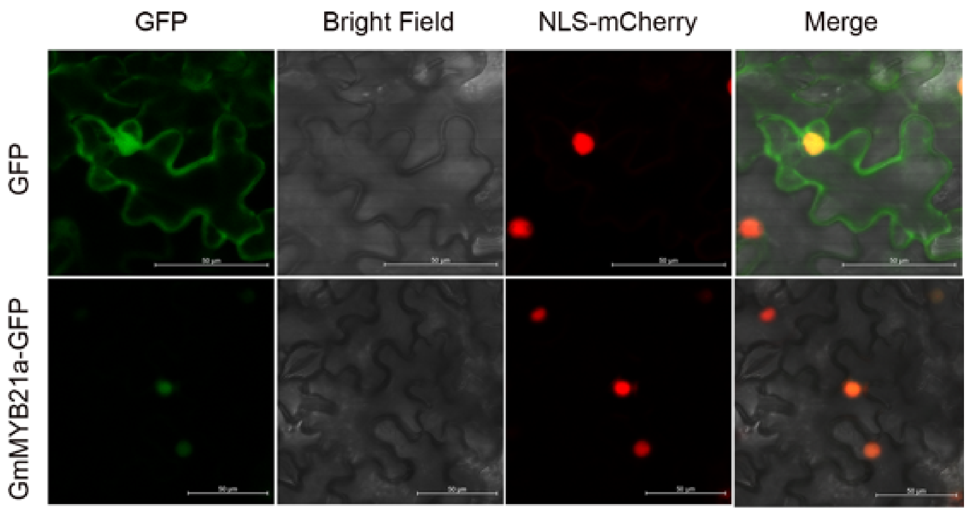

Supplementary Figure S3. The subcellular localization of GmMYB21a in *N. benthamiana* leaves. The NLS-mCherry acted as the nuclear marker. The GFP was used as the control. Bar, 50 μm.

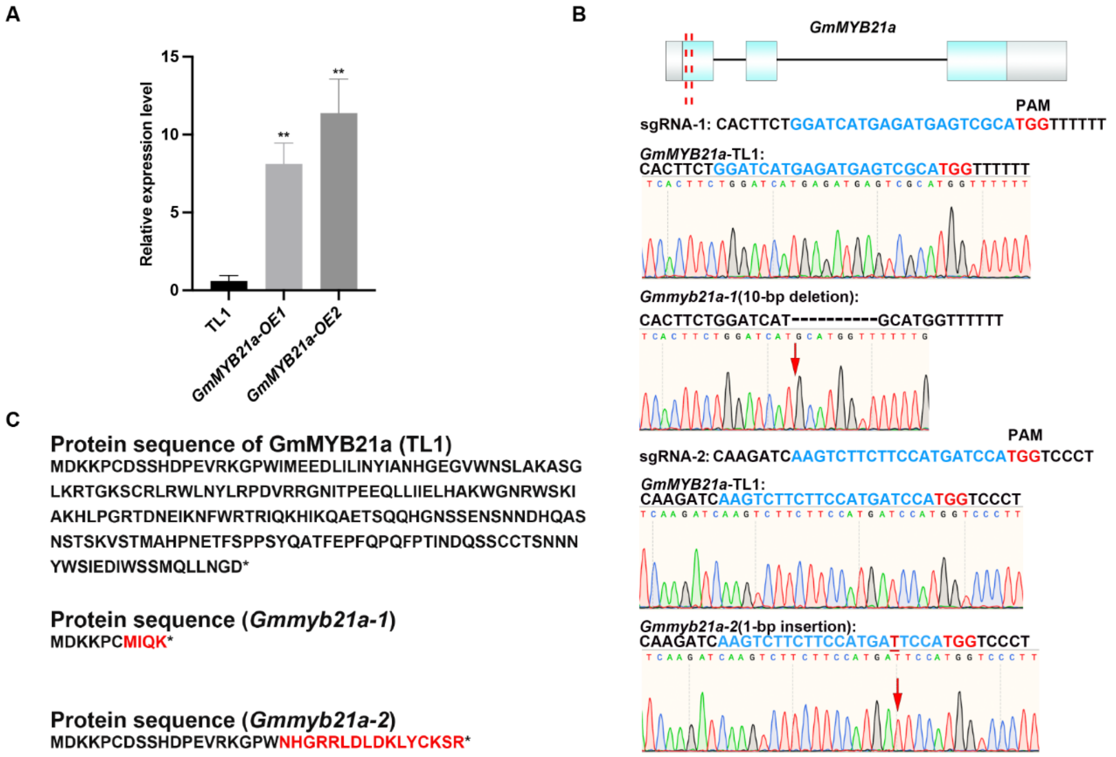

Supplementary Figure S4. Identification of *GmMYB21a* overexpression lines and *Gmmyb21a* mutants. (a) The relative expression level of *GmMYB21a* in *GmMYB21a-OE1* and *GmMYB21a-OE2*. *GmActin11* acted as an internal reference gene. Data were shown as mean ± SD from three biological replicates. Asterisks indicated significant differences according to Student's *t*-test (**, $P < 0.01$). (b) Scheme of the CRISPR (Clustered Regularly Interspaced Short Palindromic Repeats)-Cas9 mediated mutation of *GmMYB21a*. The blue fonts indicated the target sites. The red arrows showed the position of mutation sites. (c) Comparison of amino acid sequence between *Gmmyb21a* mutants and TL1. Red fonts represented mutated amino acids.
